# The role of EII complex in the bacterial responses to the glucose-survey in clinical *Klebsiella pneumoniae* isolates

**DOI:** 10.1371/journal.pone.0289759

**Published:** 2023-08-07

**Authors:** Yu-Tze Horng, Novaria Sari Dewi Panjaitan, Yi-Jhen Tsai, Pin-Wei Su, Hung-Chi Yang, Po-Chi Soo

**Affiliations:** 1 Department of Laboratory Medicine and Biotechnology, College of Medicine, Tzu Chi University, Hualien, Taiwan, R.O.C; 2 Center for Biomedical Research, Research Organization for Health, National Research and Innovation Agency (BRIN), Cibinong Science Center, Cibinong, Bogor, West Java, Indonesia; 3 Department of Medical Laboratory Science and Biotechnology, Yuanpei University of Medical Technology, Hsinchu, Taiwan, R.O.C; BOKU: Universitat fur Bodenkultur Wien, AUSTRIA

## Abstract

Type 3 fimbriae in *Klebsiella pneumoniae* are important for bacterial colonization on abiotic and biotic surfaces. The major subunit of type 3 fimbriae (MrkA) is increased by overexpression of EtcABC, an EII complex of phosphoenolpyruvate:carbohydrate phosphotransferase systems (PTSs), through cAMP-cAMP receptor protein (cAMP-CRP) in *K*. *pneumoniae* STU1. Here, we further characterized the relations between the amount of *etcABC* mRNA and MrkA in 78 clinical *K*. *pneumoniae* isolates incubated in high levels of glucose. By Western blotting, we observed that MrkA of 29 isolates were not decreased much by high levels of glucose (Group A) but MrkA of other 49 isolates were significantly reduced (Group B) in the same condition. The bacterial biofilms on abiotic surfaces and colonization in the *Caenorhabditis elegans* of representative isolates in the Group A were not affected by high levels of glucose. However, the biofilm and colonization in the worm of clinical isolates in the Group B were much reduced by high levels of glucose. After quantification by real time RT-PCR, 76% of Group A but just 10% of Group B showed high amount of *etcA* mRNA. In summary, our results suggested that for most of *K*. *pneumoniae* clinical isolates, the amount of *etcABC* mRNA was positively related to their type 3 fimbriae production in a high level of glucose, thereby to their biofilm formation and colonization in the worm.

## Introduction

*Klebsiella pneumoniae* can be found ubiquitously in natural superficial waters and soils [1] and colonize in the gastrointestinal tracts of humans and animals [2–4]. *K*. *pneumoniae* can be detected in 62.1% of stool specimens from healthy adults [5]. In addition, *K*. *pneumoniae* can cause opportunistic infections, including urinary tract infections and pneumonia [1, 6, 7]. Human guts are reservoirs of hypervirulent *K*. *pneumoniae* [8]. *K*. *pneumoniae* forms biofilms on catheters or ventilators, increasing the risk of urinary tract infections, bloodstream infections, and pneumonia [9]. *K*. *pneumoniae* can also form biofilms on tissue surfaces. Furthermore, *K*. *pneumoniae* adheres to the surfaces mainly via type 1 and type 3 fimbriae [1]. Most members of the Enterobacteriaceae family express type 1 fimbriae, but just several members of this family produce type 3 fimbriae [10]. In *K*. *pneumoniae*, type 3 fimbriae are synthesized by proteins encoded by the *mrkABCDF* operon [11]. The major subunits of the type 3 fimbrial shaft are encoded by the *mrkA* gene, while MrkD is an adhesin of type 3 fimbriae that facilitates bacterial binding to the collagen-coated surface or extracellular matrix formed by bronchial cells. In addition, type 3 fimbriae promote the colonization of *K*. *pneumoniae* on abiotic surfaces to form biofilms [10, 12]

To translocate various sugars, bacteria possess several phosphoenolpyruvate (PEP): carbohydrate phosphotransferase systems (PTSs) to respond to carbohydrate availability. The phosphate group is sequentially transferred from PEP to the related carbohydrates via PTS, which generally consists of enzyme I (EI), histidine-containing phosphocarrier protein (HPr) and enzyme II (EII) complexes. EI and HPr are general to all PTSs, but EII complexes are sugar-specific. EII complexes are composed of EIIA, EIIB and EIIC (and sometimes EIID) proteins/domains. The membrane-bound EIIC is directly responsible for translocating sugar into bacteria. The phosphate group from HPr is transferred to EIIA, then to EIIB and eventually to the sugar in the cytoplasm [13, 14]. In addition to carbohydrate transportation, EIIA and EIIB are also involved in various physiological processes in bacteria [15]. For example, Crr, which is a glucose-specific EIIA in enteric bacteria, interacts with glycerol kinase, leading to inhibition of glycerol uptake in the presence of glucose. In addition, Crr regulates the synthesis of cAMP via interactions with adenylate cyclase (AC) [13, 16]. We reported that EtcA, EtcB and EtcC in *K*. *pneumoniae* STU1 (homologous to KPN00353, KPN00352 and KPN00351 in *K*. *pneumoniae* MGH78578, respectively) were novel EIIA, EIIB, and EIIC homologs respectively and encoded from genes in an operon, *etcABC* [17]. Overexpression of *etcABC* increased biofilm and type 3 fimbriae in *K*. *pneumoniae* [18, 19]. Deficiency of *crr* in *K*. *pneumoniae* STU1 resulted in a low level of cAMP production, but overexpression of *etcABC* elevated the cAMP level in the Δ*crr*/Δ*etcABC* double mutant [19]. The capsular polysaccharide (CPS) of the *crr* mutant is higher than that of the wild type, but deletion of *etcABC* in the *crr* mutant decreases CPS synthesis [20].

We previously reported that EtcABC regulated the type 3 frimbriae in *K*. *pneumonniae* STU1 via cAMP-cAMP receptor protein (CRP) [19]. Given literature on the activity of CRP regulated by the glucose-specific EIIA in other bacteria [16], we identified the relation between glucose, *etcABC* and type 3 frimbriae in *K*. *pneumoniae* in this study. Since the result from a single strain may not represent the phenomena of most clinical strains, 78 clincal *K*. *pneumoniae* isolates were examined in this study.

## Materials and methods

### *K*. *pneumoniae* strains and growth conditions

*K*. *pneumoniae* STU1 is a laboratory-maintained strain that was acquired from National Taiwan University (Taipei, Taiwan). Seventy-eight *K*. *pneumoniae* clinical strains were isolated from clinical specimens (sputum, blood, wound, urine and others) during 2016–2018 in Tzu Chi Hospital (Hualien, Taiwan) and then transferred to Tzu Chi University (Hualien, Taiwan) through an official transfer. *K*. *pneumoniae* STU1/pBSK-Km::ZsGreen, which carries the ZsGreen gene to produce green fluorescence, was constructed in our previous study [20]. *K*. *pneumoniae* Clin200 and *K*. *pneumoniae* Clin73, two of the 78 clinical isolates mentioned above, were isolated from sputum specimens. In this study, the plasmid pBSK-Km::ZsGreen was transformed into *K*. *pneumoniae* Clin200 and *K*. *pneumoniae* Clin73 to emit green fluorescence according to methods described previously [20]. *K*. *pneumoniae* STU1/pBSK::Gm::etcABC, which carries the promoter and structure of *etcABC* in the plasmids to overexpress *etcABC*, and *K*. *pneumoniae* STU1/pBSK::Gm, which was the vector control in this study, were constructed in our previous study [19]. *K*. *pneumoniae* was routinely cultured in Luria-Bertani (LB) medium (0.5% yeast extract, 1% tryptone and 1% NaCl) at 37°C. For the bacteria carrying pBSK-Km::ZsGreen, pBSK, pBSK::Gm or pBSK::Gm::etcABC, LB was supplemented with 50 μg/mL kanamycin or 20 μg/mL gentamicin in routine culture.

### Nematode maintenance

*Caenorhabditis elegans* N2 (wild type) was a kind gift from Professor Hung-Chi Yang at the Department of Medical Laboratory Science and Biotechnology, Yuanpei University of Medical Technology and was propagated on a nematode growth medium (NGM) plate seeded with *Escherichia coli* OP50 prior to being fed with *K*. *pneumoniae* [21].

### Observation of bacteria in the intestine of a nematode

After bacteria with pBSK-Km::ZsGreen were cultured in LB with 50 μg/mL kanamycin at 37°C overnight, the bacteria were resuspended in fresh LB to an OD_600_ of 9. Fifty microliters of this bacterial suspension were pipetted onto NGM plates without or with 2% glucose. Then, approximately 50~60 *C*. *elegans* were cultured on these plates for 3 days. After moving from the plates to microscope glass slides, the worms were washed with 0.9% NaCl three times. Subsequently, the *C*. *elegans* was paralyzed with 200 mM sodium azide for 5~10 min. Images of nematodes were captured using an upright fluorescence microscope Nikon Ni-E (Nikon, Japan). In order to quantify the fluorescence levels in worms, 20 *C*. *elegans* in a well of microplate (PerkinElmer, USA) were detected at 505 nm after 492-nm excitation by Varioskan Flash microplate reader (Thermo Fisher Scientific, USA).

### Western blotting

After overnight culture in LB, bacteria were diluted in LB containing 0%, 0.1%, 0.2%, 0.5%, 1% or 2% glucose respectively to optical density at 600 nm (OD_600_) of 0.08 and then incubated for 24 hours at 37°C. After determination of bacterial concentration by measuring their OD_600_, the bacteria were suspended in the SDS sample buffer and lysed at 100°C for 10 min. An aliquot of cell lysis was applied to 12% SDS-PAGE, following the procedures by Sambrook [22]. The MrkA and ManA (mannose 6-phosphate isomerase), were detected using Western blotting according to our previous study [19]. ManA was used as the loading control in Western blotting. The Western blotting signals were detected using the Gel Catcher 2850 chemiluminescence camera system (CLUBIO, Taipei, Taiwan) and analyzed by ImageJ (National Institutes of Health).

### Quantification of biofilm

The bacterial biofilms formed in LB without/with 2% glucose for 24 hours at 37°C were quantified according to our previous study [19].

### Reverse transcription quantitative real-time PCR (RT-qPCR)

The transcriptional levels of *etcA* and *recA* were quantified by RT-qPCR as previously described and normalized to that of 16S rRNA following the 2^−ΔΔCT^ method [20]. The *recA*, a housekeeping gene encoding recombinase A, was used as a reference to compare the gene expression of *etcA*. All primers and probes are listed in Table 1.

**Table 1:** Oligonucleotide primers and probes for RT-qPCR in this study.

Primer/probe	Sequences (5’—3’)	Target
KPN353 FP	TTCGCCCTACCTCTCACTTT	*etcA*
KPN353 RP	CCGTTACCACAAACCGTTCT	*etcA*
etcA probe	/56-FAM/TTATCACCA/ZEN/ CAGCCACGCCAATCT/3IABkFQ/	*etcA*
recA qPCR FP	CCGCTTTCTCAATCAGCTTC	*recA*
recA qPCR RP	TTAAACAGGCCGAATTCCAG	*recA*
recA probe	/56-FAM/TCGCCGTAG/ZEN/ AAGTTGATGCCTTCG/3IABkFQ/	*recA*
RNA 16S qPCR FP	ATGACCAGCCACACTGGAAC	16S rRNA
RNA 16S qPCR RP	CTTCCTCCCCGCTGAAAGTA	16S rRNA
16S probe	/56-FAM/ATGTCTGGG/ZEN/ AAACTGCCTGATGGA/3IABkFQ/	16S rRNA

### Statistical analysis

For RT-qPCR and quantification of biofilm and fluorescence, the values were expressed as the average +/- standard deviation (SD) from three independent experiments. Data were subjected to analysis using a student’s t-test. Significant difference was considered at *p* < 0.05.

## Results

### Discrepancy in the effects of glucose on type 3 fimbriae and biofilms in *K*. *pneumoniae* clinical isolates

**Fig 1:**
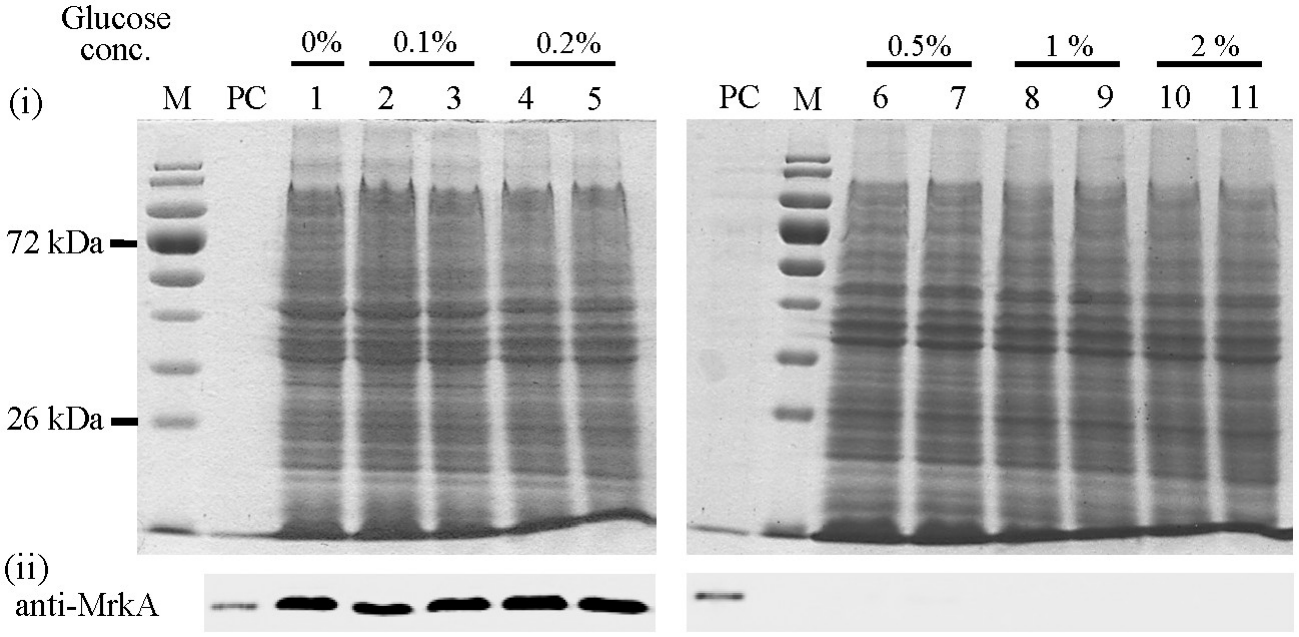
The effects of glucose on *K*. *pneumoniae* STU1. The bacteria were incubated in LB (Lane 1) or LB supplemented with glucose from 0.1% (duplicated loading on lane 2 and 3), 0.2% (lane 4 and 5), 0.5% (lane 6 and 7), 1% (lane 8 and 9) to 2% (lane 10 and 11) as the final glucose concentration. After incubation for 24 hours, the cellular proteins were analyzed by SDS-PAGE (i) and Western blotting using an antibody against MrkA (anti-MrkA) (ii). PC: MrkA (21 kDa) as a positive control. The protein marker is on the left lane of SDS‒PAGE. The SDS‒PAGE images showed consistency of loading and general pattern of cell lysate of each sample. Representative images of SDS and Western blotting were from three independent experiments.

**Fig 2:**
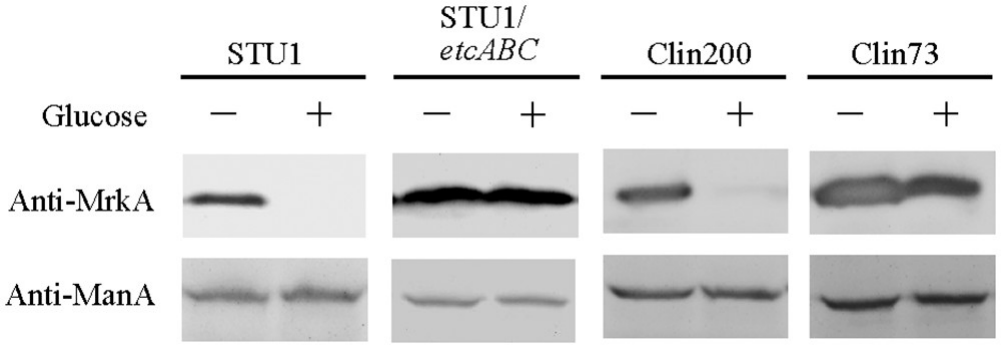
Western blotting analysis of MrkA and ManA. The bacteria were incubated in LB (-) or LB supplemented with 2% glucose (+) for 24 hours. STU1: *K*. *pneumoniae* STU1. STU1/etcABC: *K*. *pneumoniae* STU1/pBSK::Gm::etcABC; Clin200 and Clin73: clinical *K*. *pneumoniae* isolates. ManA (mannose 6-phosphate isomerase) was the loading control. Representative images were from three independent experiments.

**Fig 3:**
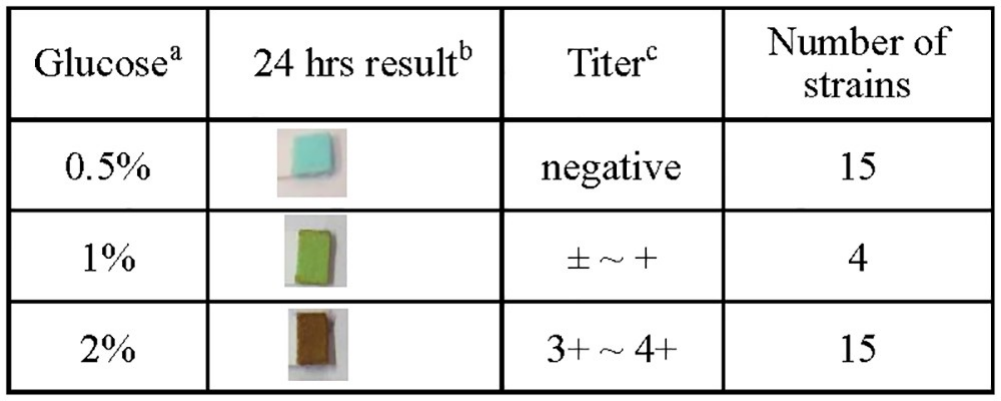
Examination of the remnant glucose in medium. (a) Fifteen *K*. *pneumoniae* clinical strains were incubated in LB medium supplemented with 0.5%, 1% or 2% glucose for 24 hours. (b) After incubation for 24 hours, the remnant glucose in medium was examined by Multistix SG reagent strips (Siemens, Germany). The images were representatives of 15, 4 and 15 clinical strains respectively in 0.5%, 1% and 2% glucose. (c) The titer of remnant glucose was determined by following the manufacturer’s instruction.

We observed that 0.5% (and more) glucose inhibited the expression of MrkA in *K*. *pneumoniae* STU1 (Figs 1 and 2). This phenomenon is inconsistent with the finding by Lin *et al*., who reported that 0.5% glucose increased MrkA expression in *K*. *pneumoniae* CG43S3 [23]. The different phenotypes of these two *K*. *pneumoniae* isolates inspired us to examine the clinical isolates. To determine which concentration of glucose cannot be exhausted by bacteria after overnight culture, 15 *K*. *pneumoniae* clinical isolates (including STU1) were incubated in LB medium supplemented with 0.5%, 1% or 2% glucose respectively. The remnant glucose in the supernatant of bacterial culture after incubation for 24 hours was examined by Multistix SG reagent strips (Siemens, Germany). The results showed that 11 clinical isolates (including STU1) depleted 1% glucose, but all 15 isolates did not exhaust the 2% glucose after culture (Fig 3). Subsequently, 2% glucose in LB was chosen to examine MrkA expression in 78 clinical isolates. By Western blotting, MrkA expression in 29 clinical isolates incubated in LB with 2% glucose were not/slightly reduced, compared to those in LB (Group A in Table 2 and Clin73 is shown as a representative in Fig 2). In contrast, MrkA expression in the other 49 bacteria incubated in LB with 2% glucose was less than half of their MrkA expression in LB (Group B in Table 2 and Clin200 is shown as a representative in Fig 2). Thus, we found that glucose reduced the expression of MrkA in 63% of clinical isolates but did not decrease MrkA expression in the other clinical isolates (37%).

**Table 2:** The number of clinical isolates tested in Western blotting and RT-qPCR.

*Klebsiella pneumoniae* clinical isolates (n = 78)^a^	
MrkA was not reduced in LB-glucose^b^ (Group A; n = 29)^a^	MrkA was reduced in LB-glucose^c^ (Group B; n = 49)^a^
High mRNA amount of *etcA*^d^^,^^f^ 76% (22/29)^a^	High mRNA amount of *etcA*^d^^,^^e^^,^^f^ 10% (2/20)^a^

Note:

^a^ The numbers in the parentheses are the amount of isolates.

^b^ Quantified by Western blotting, the amount of MrkA in bacterium incubated in LB with 2% glucose was more than half of that in bacterium in LB.

^c^ Quantified by Western blotting, the amount of MrkA in bacterium incubated in LB with 2% glucose was less than half of that in bacterium in LB.

^d^ The mRNA amount of *etcA* and *recA* in bacteria were quantified by RT-qPCR respectively. The *recA* is house-keeping gene and used as reference in RT-qPCR.

^e^ The 20 isolates were randomly chosen from 49 isolates in Group B.

^f^ If the ratio of *etcA* mRNA/*recA* mRNA was higher than one, the mRNA amount of *etcA* was defined as high level.

**Fig 4:**
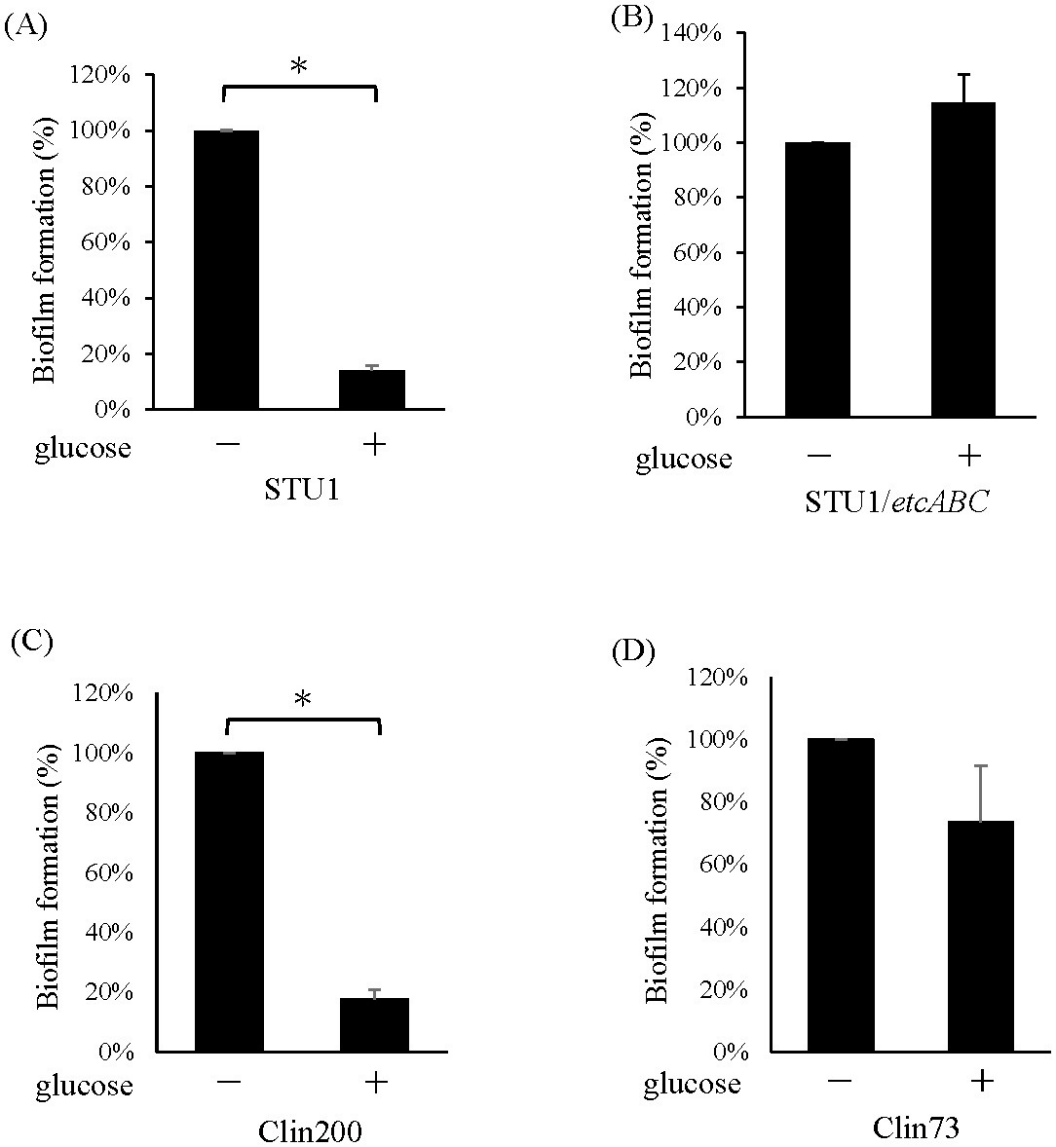
The effects of glucose on biofilm formation. The bacteria were incubated in LB (-) or LB with 2% glucose (+) for 24 hours. The amount of biofilm in LB with glucose was compared to that in LB. The error bars indicate the SD from three independent cultures. An asterisk (*) indicates that the data were significantly different at *p* < 0.05. STU1: *K*. *pneumoniae* STU1. STU1/etcABC: *K*. *pneumoniae* STU1/pBSK::Gm::etcABC, Clin200 and Clin73: clinical *K*. *pneumoniae* isolates.

Since MrkA is the major subunit of type 3 fimbriae, which promotes the attachment of bacteria to abiotic surfaces to form biofilms [1], the effects of glucose on biofilms of *K*. *pneumoniae* STU1, Clin200 and Clin73 were examined. The results showed that the biofilms of *K*. *pneumoniae* STU1 and Clin200 in LB with glucose were much less than those in LB, while the biofilm of *K*. *pneumoniae* Clin73 in LB with glucose did not decrease much, compared to that in LB (Fig 4). Thus, the effects of glucose on fimbriae and biofilms are positively correlated in one strain, but the effects of glucose on the *K*. *pneumoniae* population vary.

### The effects of glucose on bacterial colonization in *Caenorhabditis elegans*

**Fig 5:**
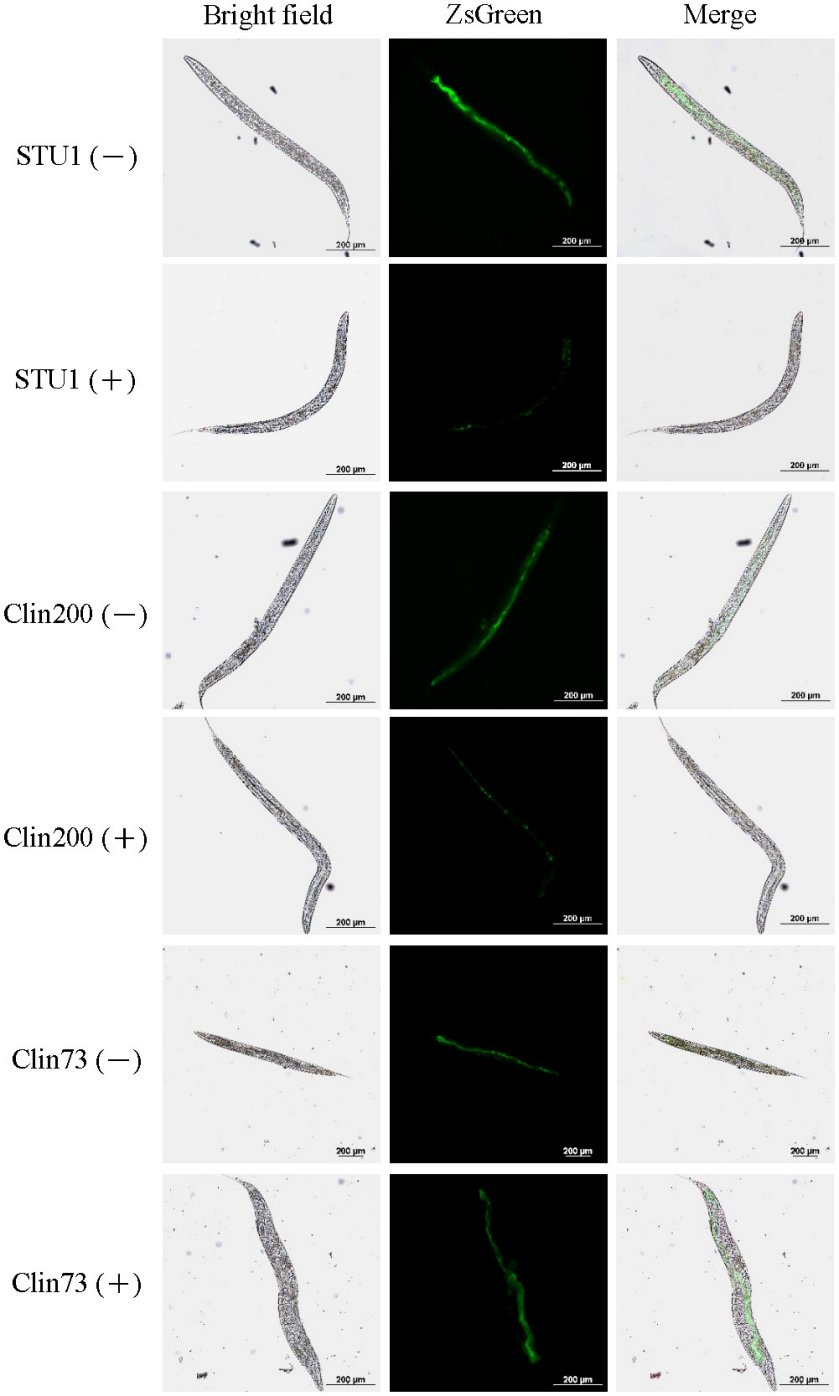
The effects of glucose on bacterial colonization in the gut of *C*. *elegans*. *K*. *pneumoniae* carrying the pBSK-Km::ZsGreen (ZsGreen) emitted fluorescence. The bacteria were seeded on NGM (-) or NGM with 2% glucose (+) to feed the *C*. *elegans*. Representative images of bacteria in the gut of nematodes were acquired by fluorescence microscopy from three independent experiments. STU1: *K*. *pneumoniae* STU1. Clin200 and Clin73: clinical *K*. *pneumoniae* isolates.

**Fig 6:**
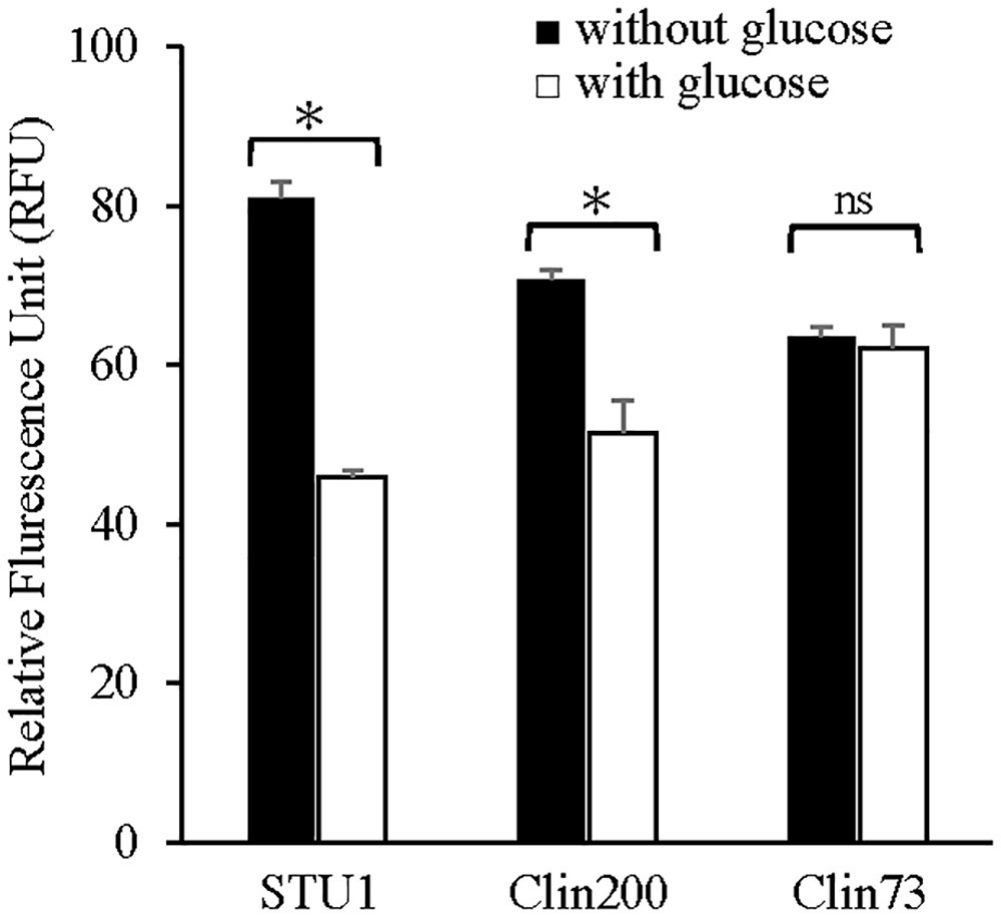
Quantification of bacteria in the gut of *C*. *elegans*. The bacteria carrying the pBSK-Km::ZsGreen were seeded on NGM (black bar) or NGM with 2% glucose (white bar) to feed the *C*. *elegans*. The fluorescence levels of 20 worms were quantified and then expressed as the average + SD from three independent experiments. STU1: *K*. *pneumoniae* STU1. Clin200 and Clin73: clinical *K*. *pneumoniae* isolates. An asterisk (*) indicates that the data were significantly different at *p* < 0.05. ns indicates non-significant difference.

Little is known about the effects of glucose on the colonization of *K*. *pneumoniae* in *Caenorhabditis elegans*. To directly observe the bacteria in the gut of worms, The plasmid, pBSK-Km::ZsGreen, was purposely transformed into *K*. *pneumoniae* STU1, Clin200 and Clin73 respectively. After *C*. *elegans* was fed a lawn of *K*. *pneumoniae* in NGM without/with 2% glucose for 3 days (Figs 5 and 6). The activity and lifespan of the worms fed with anyone of *K*. *pneumoniae* isolates (STU1, Clin200 and Clin73) were not different. However, the fluorescence intensities from *K*. *pneumoniae* STU1 and Clin200 were decreased in the nematodes from the NGM with glucose, compared to that from the NGM. In contrast, the fluorescence intensity of *K*. *pneumoniae* Clin73 in *C*. *elegans* on the NGM with glucose addition was not significantly different from that on the NGM (Figs 5 and 6). This result indicated that the colonization number of *K*. *pneumoniae* STU1 and Clin200 in *C*. *elegans* was reduced by glucose, but glucose did not affect the colonization of *K*. *pneumoniae* Clin73 in the worm. To confirm whether glucose influences *C*. *elegans*, *C*. *elegans* was fed on a lawn of *E*. *coli* OP50 on NGM without/with 2% glucose. The results showed that the activity and lifespan of the nematode were not influenced by glucose. Thus, the results showed the various bacterial responses to glucose in colonization in *C*. *elegans*, related to bacterial fimbriae production.

### High amount of *etcABC* mRNA is related to fimbriae resistance to glucose inhibition

In our previous study, overexpression of *etcABC* in *K*. *pneumoniae* STU1 enhanced type 3 fimbriae expression in the LB [19]. Furthermore, *K*. *pneumoniae* STU1/pBSK::Gm::etcABC, also showed high levels of the type 3 fimbriae in the LB with glucose, compared to its parent strain (STU1) (Fig 2). In addition, the biofilm of *K*. *pneumoniae* STU1/pBSK::Gm::etcABC was not decreased in the LB with glucose, compared with that in the LB (Fig 4B). We did not transform pBSK-Km::ZsGreen into the *K*. *pneumoniae* STU1/pBSK::Gm::etcABC to observe it in the gut of the nematode because of the same replication origin of two plasmids.

**Fig 7:**
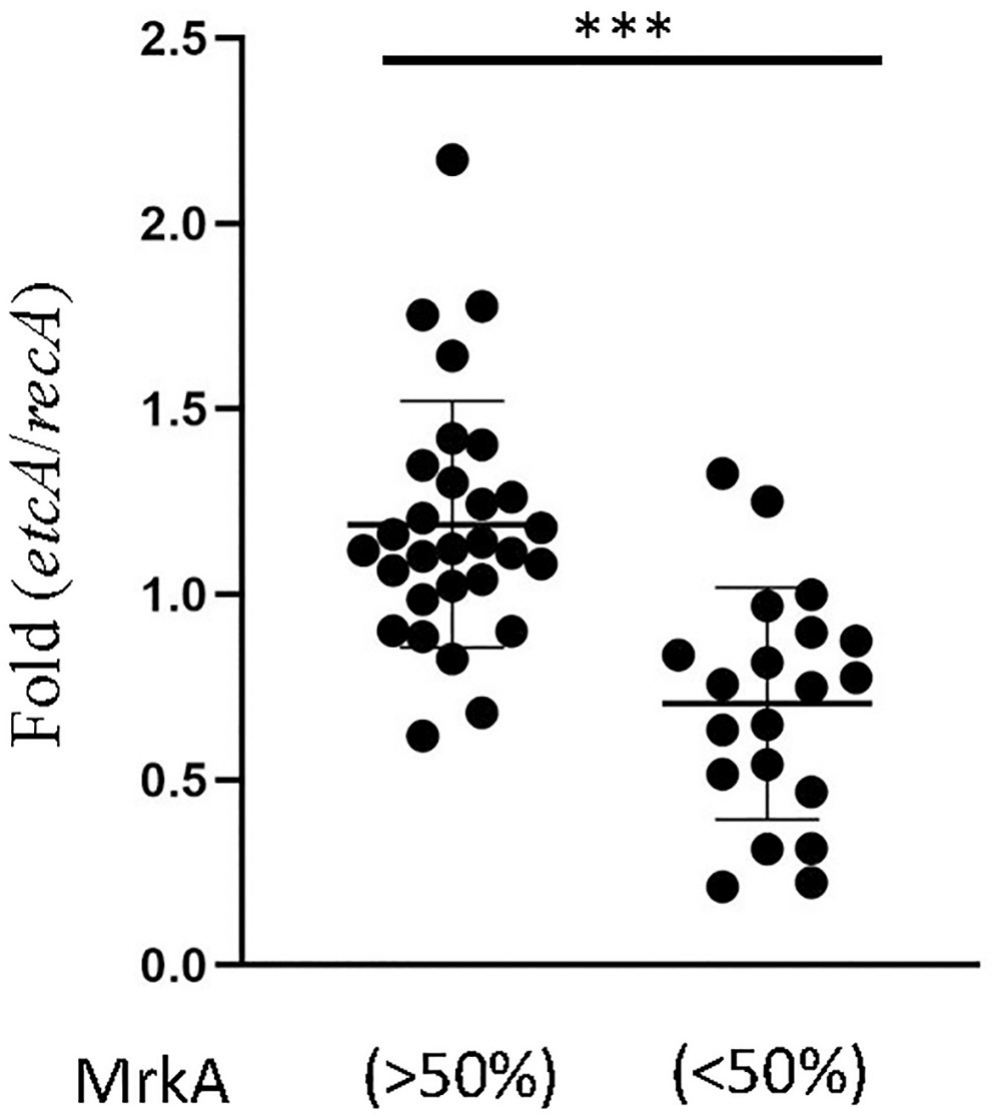
RT‒qPCR analysis of the *etcA* and *recA* transcripts in clinical isolates. The mRNA level of *etcA* was compared to that of *recA*, a housekeeping gene encoding recombinase A, in each *K*. *pneumoniae* isolate. MrkA>50%: the 29 clinical isolates in Group A. MrkA<50%: the 20 clinical isolates randomly selected from Group B. Each dot represents one clinical isolate. The error bars indicate SD from the population. Asterisks (***) represent *p* < 0.05.

Herein, we hypothesized that the clinical bacteria in Group A had high mRNA levels of *etcABC*, resulting in their high production of type 3 fimbriae in the medium with glucose. Therefore, the amount of *etcA* mRNA in clinical isolates was quantified to examine this hypothesis since *etcABC* was transcribed as an operon [17]. After RT‒qPCR was performed, the amount of *etcA* mRNA was normalized to that of *recA* mRNA, which is a housekeeping gene, in an individual clinical isolate. If the ratio of *etcA* to *recA* was higher than one, the amount of *etcA* mRNA was defined as high level. The results showed that high levels of *etcA* mRNA were found in 22 isolates in Group A (76%, 22/29). However, 20 isolates were randomly selected from Group B to be tested and showed that 90% (18/20) isolates in Group B expressed low amount of *etcA* mRNA (Table 2 and Fig 7). The results indicated that amount of *etcABC* mRNA was positively related to type 3 fimbriae production of *K*. *pneumoniae* in the high concentration of glucose.

## Discussion

Through analyzing clinical *K*. *pneumoniae* isolates, the various effects of glucose on *K*. *pneumoniae* were observed in this study. For 37% of the clinical *K*. *pneumoniae* isolates (Group A), their MrkA was not decreased or was slightly decreased at high glucose concentrations, but MrkA in 63% of the clinical isolates (Group B) was reduced by glucose (Table 2). Most strains in Group A also showed high amount of *etcA* mRNA. In contrast, most strains in Group B showed low amount of *etcA* mRNA (Table 2 and Fig 7). The *K*. *pneumoniae* isolate Clin73, one of GroupA, also presented a high level of biofilm on the abiotic surface and colonization in the gut of worms in the medium with glucose, compared to that without glucose (Figs 4–6). In general, the effects of glucose on the expression of type 3 fimbriae, biofilm formation and gut colonization in the worm were positively related to the amount of *etcABC* mRNA in the most of *K*. *pneumoniae* isolates.

**Fig 8:**
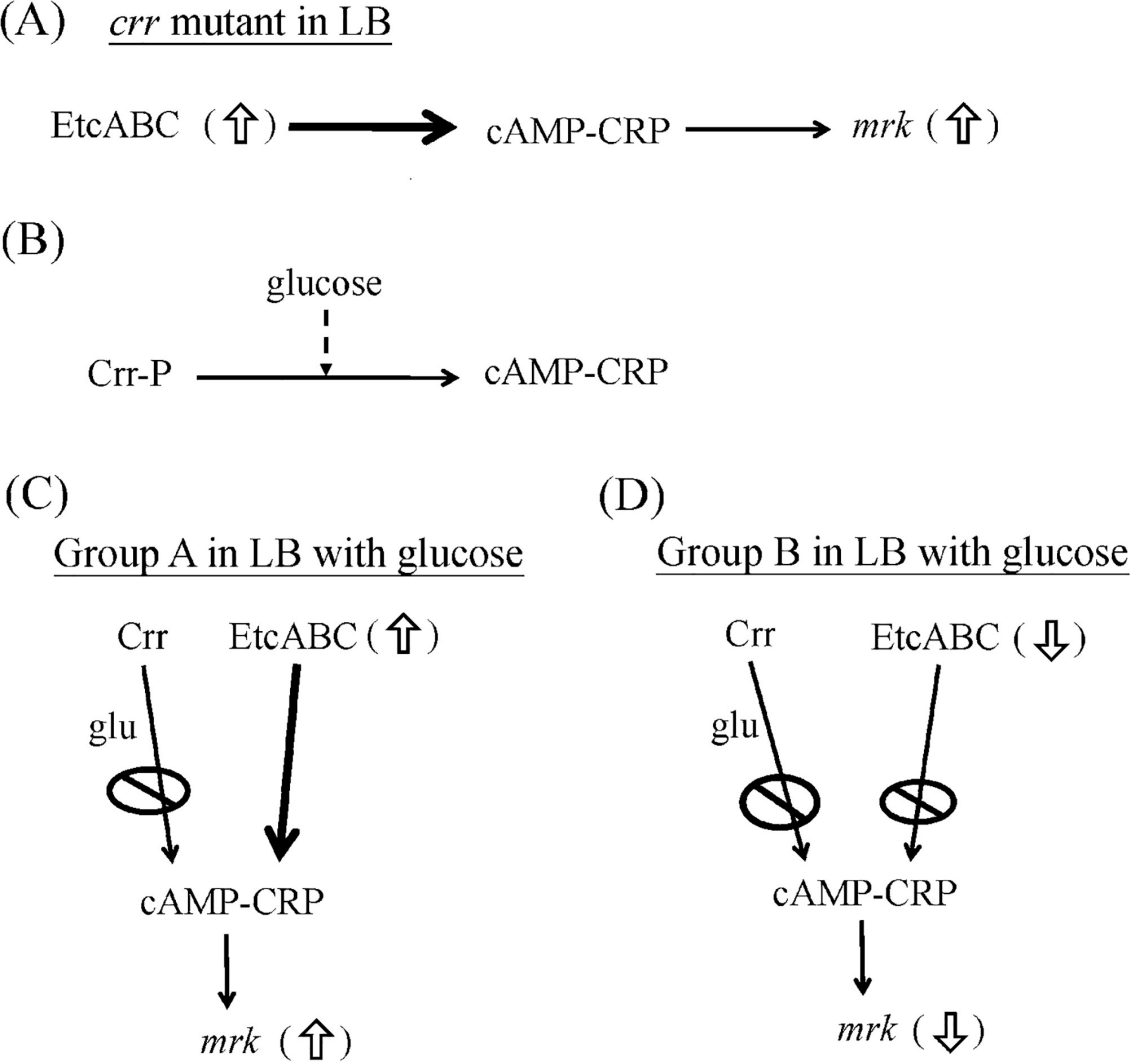
Schematic diagram of the roles of EtcABC in type 3 fimbriae production by *K*. *pneumoniae* growing in the medium containing glucose. The possible regulatory mechanisms are proposed by combination of the previous research and this study. (A) Overexpression of EtcABC in *K*. *pneumoniae crr* mutant increased the activity of cAMP-CRP and then *mrk* operon, resulting the high production of type 3 fimbriae in LB [19]. (B) In *E*. *coli* and several members in Enterobacteriaceae, the activity of cAMP-CRP is increased by Crr in phosphorylated state but inhibited by glucose due to dephosphorylation of Crr in the environment with glucose [16]. (C) In 76% of Group A growing in the LB containing glucose, high amount of *etcABC* mRNA (EtcABC) activates cAMP-CRP and then *mrk* operon is increased. (D) In 90% of Group B growing in the LB containing glucose, Crr in unphophorylated state and EtcABC in low amount cannot activate cAMP-CRP, leading to decreased expression of *mrk* operon and then type 3 fimbriae production. Crr-P: phosphorylated Crr. *mrk*: *mrk* operon. Dashed arrow: inhibition. Black arrow: activation. White arrow: expression levels of genes are increased (up) or decreased (down). Forbidden sign: the gene regulatory activity is inhibited or reduced. glu: glucose.

We previously reported that *etcABC* operon is wildly distributed in the most of *K*. *pneumoniae* isolates [17]. Overexpression of EtcABC increased the amount of cAMP in *K*. *pneumoniae* STU1. The high level of cAMP caused CRP to increase the transcription of the *mrk* operon, leading to an increase in type 3 fimbriae production (Fig 8A) [19]. In addition, high concentration of glucose results in low amount of intracellular cAMP in *E*. *coli*, which does not have *etcABC* (Fig 8B) [16, 24]. Taken together, we speculated that high concentration of the glucose led to inactivation of Crr (unphosphorylated Crr) and the amount of *etcABC* mRNA and cAMP levels in 90% of clinical isolates in Group B were low, resulting in reduced MrkA in the bacteria growing in the medium containing high concentration of glucose (Fig 8D). In contrast, even growing in the high concentration of glucose, the 76% of clinical isolates in Group A expressed high amount of *etcABC* mRNA and cAMP-CRP, resulting in high expression of type 3 fimbriae (Fig 8C).

Thus, we hypothesized that the upstream DNA regions of *etcABC* may affect the transcriptional level of *etcABC* in various *K*. *pneumoniae* clinical isolates. Then, we examined 400-bp DNA region upstream of *etcABC* in several clinical isolates and chose some of them to be ligated with the reporter gene, *luxCDABE* [19]. By measuring the bioluminescence levels from the reporter, the results showed that the transcriptional activities from these 400-bp regions were not different. Therefore, the 400-bp upstream region of *etcABC* is not the factor in 2% glucose to reduce *etcABC* transcription in Group B or keep *etcABC* transcription high in Group A.

Lin *et al*. reported that MrkA in *K*. *pneumoniae* CG43S3 was increased in LB with 0.25%-0.5% glucose, compared to that in LB [23]. Although we did not examine *K*. *pneumoniae* CG43S3, any clinical isolate in this study did not show that MrkA was increased in the LB with 2% glucose, compared to that in LB. In addition, 0.25%-0.5% glucose is inferred to be exhausted by bacteria after culture (Fig 3). Therefore, the model of MrkA regulation by glucose that proposed by Lin *et al*. is rarely applicable in clinical *K*. *pneumoniae* isolates. However, the report by Lin *et al*. also demonstrated that the biofilm of *K*. *pneumoniae* CG43S3 in LB with 0.5%-2% glucose was less than that in LB, similar to *K*. *pneumoniae* STU1 and *K*. *pneumoniae* Clin200 in Fig 4.

A retrospective study reported that 44% of patients with *K*. *pneumoniae* bacteremia in Taiwan in 2014 and 2016 were diabetes mellitus (DM) patients [25]. From the findings in this study, we think that high amount of *etcABC* mRNA and type 3 fimbriae of *K*. *pneumoniae* resistance to glucose may play important roles in DM patients that suffer from infection. Therefore, further study on high-*etcABC*-mRNA *K*. *pneumoniae* in DM patients is needed in the future. In addition, DM was defined as a fasting blood glucose level of 126 mg/dL or higher on two separate tests. (World Health Organization. https://www.who.int/data/gho/indicator-metadata-registry/imr-details/2380). The 2% glucose concentration in this study was much higher than the blood glucose concentration. However, glucose was gradually consumed by *K*. *pneumoniae* in our experiments. The bacteria may change their metabolic status after glucose was depleted. To maintain *K*. *pneumoniae* in the medium with glucose, we chose 2% glucose in this study (Fig 3). In future studies, continuous fermentation to maintain glucose at a certain concentration can be performed to examine the expression of type 3 fimbriae in *K*. *pneumoniae*.

In this study, we reported that the expression of type 3 fimbriae in clinical *K*. *pneumoniae* isolates growing in the medium with glucose were various and related to the amount of *etcABC* mRNA. The effects of glucose on bacterial biofilm formation and colonization in the gut of worm are consistent with the expression of type 3 fimbriae, depending on *etcABC*, in the most of *K*. *pneumoniae* isolates.

## Supporting information

S1 Raw image The effects of glucose on *K*. *pneumoniae* STU1. The bacteria were incubated in LB (Lane 1) or LB supplemented with glucose from 0.1% (lane 2 and 3), 0.2% (lane 4 and 5), 0.5% (lane 6 and 7), 1% (lane 8 and 9) to 2% (lane 10 and 11) as the final glucose concentration. The cellular proteins were analyzed by SDS-PABE (A and B). PC means MrkA (21 kDa) as a positive control. The protein markers (M) shows 170 kDa, 130 kDa, 95 kDa, 72 kDa, 55 kDa, 43 kDa, 34 kDa, 26 kDa. The raw images of Western blotting from chemiluminescence camera system (C and D) are inverted to (E and F) by inverting black and white.
(PDF)

S2 Raw image Western blotting analysis of MrkA and ManA. The raw images of Western blotting from chemiluminescence camera system (A) are inverted to (B) by inverting black and white. (-) means LB and (+) means LB supplemented with 2% glucose. STU1: *K*. *pneumoniae* STU1. STU1/etcABC: *K*. *pneumoniae* STU1/pBSK::Gm::etcABC; Clin200 and Clin73: clinical *K*. *pneumoniae* isolates.
(PDF)
