# Supplementary material for: The role of EII complex in the bacterial responses to the glucose-survey in clinical Klebsiella pneumoniae isolates
Source: PLoS One. 2023 Aug 7;18(8):e0289759. doi: 10.1371/journal.pone.0289759 (PMC10406186; doi:10.1371/journal.pone.0289759)
Supplement: S1 Raw image — The bacteria were incubated in LB (Lane 1) or LB supplemented with glucose from 0.1% (lane 2 and 3), 0.2% (lane 4 and 5), 0.5% (lane 6 and 7), 1% (lane 8 and 9) to 2% (lane 10 and 11) as the final glucose concentration. The cellular proteins were analyzed by SDS-PABE (A and B). PC means MrkA (21 kDa) as a positive control. The protein markers (M) shows 170 kDa, 130 kDa, 95 kDa, 72 kDa, 55 kDa, 43 kDa, 34 kDa, 26 kDa. The raw images of Western blotting from chemiluminescence camera system (C and D) are inverted to (E and F) by inverting black and white. (PDF) [file pone.0289759.s001.pdf]

Supporting information: Raw image of figure 1

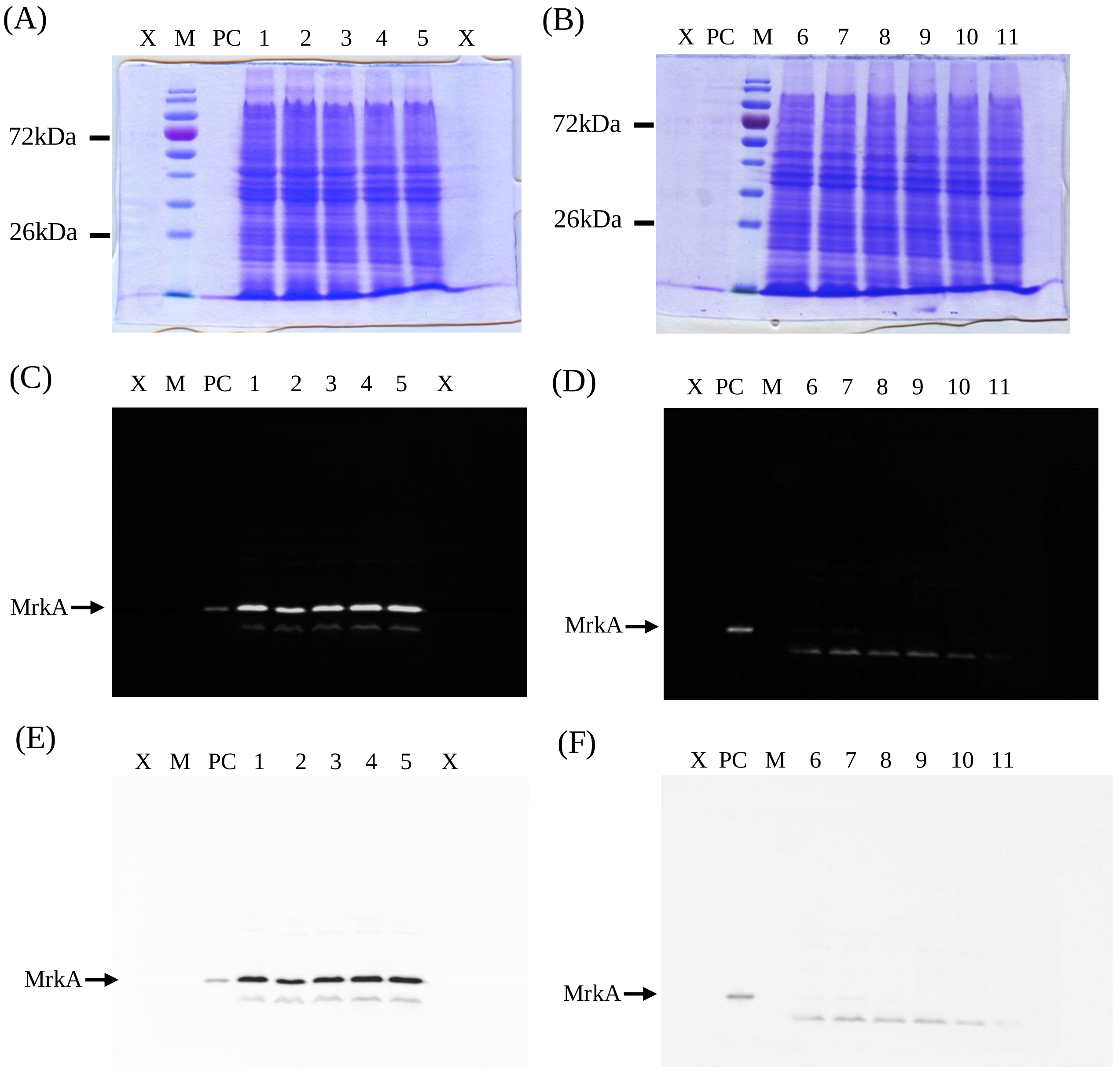

S1 Fig. The effects of glucose on *K. pneumoniae* STU1. The bacteria were incubated in LB (Lane 1) or LB supplemented with glucose from 0.1% (lane 2 and 3), 0.2% (lane 4 and 5), 0.5% (lane 6 and 7), 1% (lane 8 and 9) to 2% (lane 10 and 11) as the final glucose concentration. The cellular proteins were analyzed by SDS-PABE (A and B). PC means MrkA (21 kDa) as a positive control. The protein markers (M) shows 170 kDa, 130 kDa, 95 kDa, 72 kDa, 55 kDa, 43 kDa, 34 kDa, 26 kDa. The raw images of Western blotting from chemiluminescence camera system (C and D) are inverted to (E and F) by inverting black and white.
