# Supplementary material for: The role of EII complex in the bacterial responses to the glucose-survey in clinical Klebsiella pneumoniae isolates
Source: PLoS One. 2023 Aug 7;18(8):e0289759. doi: 10.1371/journal.pone.0289759 (PMC10406186; doi:10.1371/journal.pone.0289759)
Supplement: S2 Raw image — The raw images of Western blotting from chemiluminescence camera system (A) are inverted to (B) by inverting black and white. (-) means LB and (+) means LB supplemented with 2% glucose. STU1: K. pneumoniae STU1. STU1/etcABC: K. pneumoniae STU1/pBSK::Gm::etcABC; Clin200 and Clin73: clinical K. pneumoniae isolates. (PDF) [file pone.0289759.s002.PDF]

Figure 2

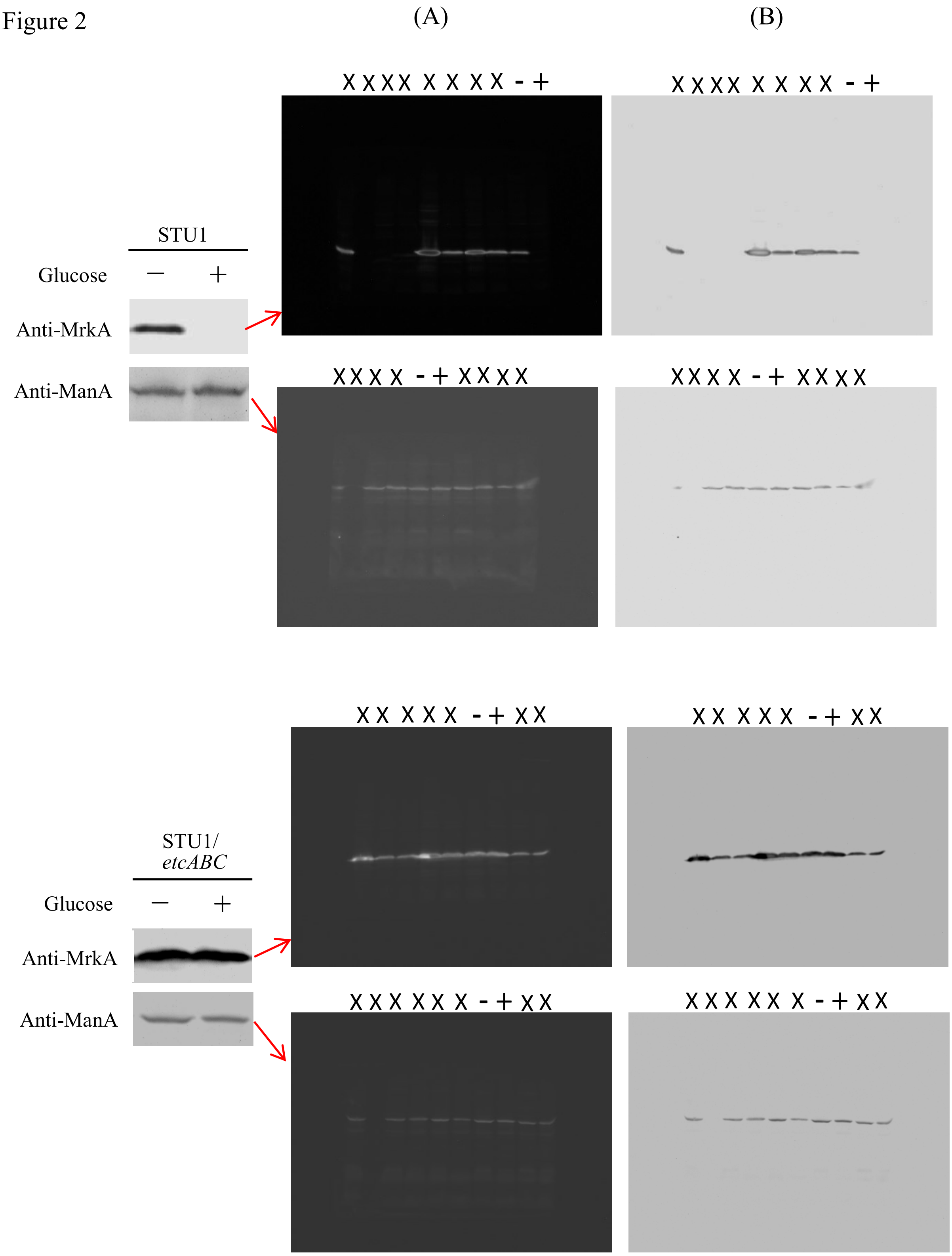

(A)
(B)
X X X X X X X X X - +
X X X X X X X X X - +
STU1
Glucose
−
+
Anti-MrkA
Anti-ManA
X X X X - + X X X X
X X X X - + X X X X
X X X X X - + X X
X X X X X - + X X
STU1/
etcABC
Glucose
−
+
Anti-MrkA
Anti-ManA
X X X X X X X - + X X
X X X X X X X - + X X

Figure 2

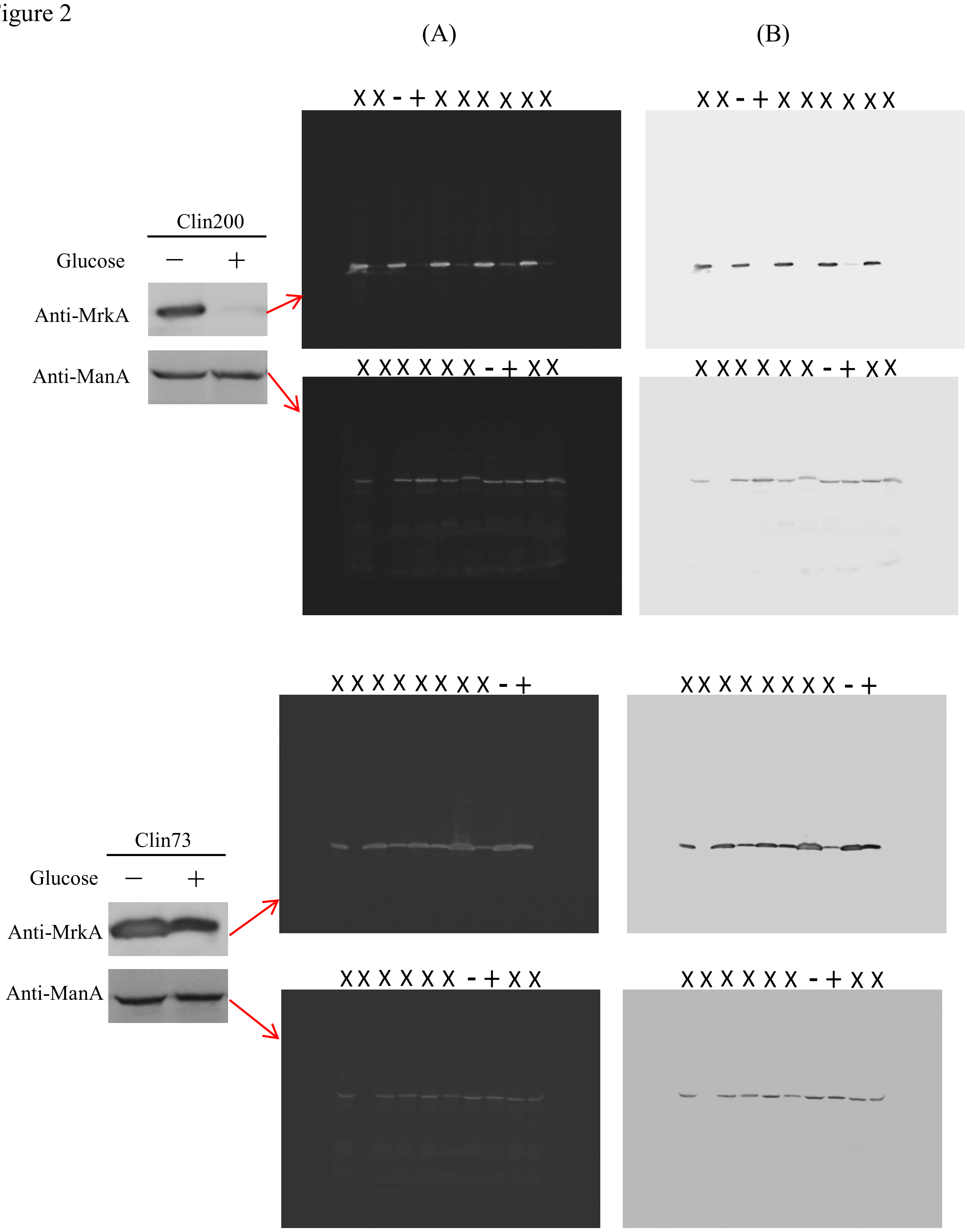

Fig 2. Western blotting analysis of MrkA and ManA. The raw images of Western blotting from chemiluminescence camera system (A) are inverted to (B) by inverting black and white. (-) means LB and (+) means LB supplemented with 2% glucose. STU1: *K. pneumoniae* STU1. STU1/etcABC: *K. pneumoniae* STU1/pBSK::Gm::etcABC; Clin200 and Clin73: clinical *K. pneumoniae* isolates.
